# Loss of Population Levels of Immunity to Malaria as a Result of Exposure-Reducing Interventions: Consequences for Interpretation of Disease Trends

**DOI:** 10.1371/journal.pone.0004383

**Published:** 2009-02-09

**Authors:** Azra C. Ghani, Colin J. Sutherland, Eleanor M. Riley, Chris J. Drakeley, Jamie T. Griffin, Roly D. Gosling, Joao A. N. Filipe

**Affiliations:** 1 MRC Centre for Outbreak Analysis & Modelling, Department of Infectious Disease Epidemiology, Imperial College London, London, United Kingdom; 2 Department of Epidemiology & Population Health, London School of Hygiene &Tropical Medicine, London, United Kingdom; 3 Department of Infectious and Tropical Diseases, London School of Hygiene & Tropical Medicine, London, United Kingdom; 4 Department of Plant Sciences, University of Cambridge, Cambridge, United Kingdom; Hong Kong University, Hong Kong

## Abstract

**Background:**

The persistence of malaria as an endemic infection and one of the major causes of childhood death in most parts of Africa has lead to a radical new call for a global effort towards eradication. With the deployment of a highly effective vaccine still some years away, there has been an increased focus on interventions which reduce exposure to infection in the individual and –by reducing onward transmission-at the population level. The development of appropriate monitoring of these interventions requires an understanding of the timescales of their effect.

**Methods & Findings:**

Using a mathematical model for malaria transmission which incorporates the acquisition and loss of both clinical and parasite immunity, we explore the impact of the trade-off between reduction in exposure and decreased development of immunity on the dynamics of disease following a transmission-reducing intervention such as insecticide-treated nets. Our model predicts that initially rapid reductions in clinical disease incidence will be observed as transmission is reduced in a highly immune population. However, these benefits in the first 5–10 years after the intervention may be offset by a greater burden of disease decades later as immunity at the population level is gradually lost. The negative impact of having fewer immune individuals in the population can be counterbalanced either by the implementation of highly-effective transmission-reducing interventions (such as the combined use of insecticide-treated nets and insecticide residual sprays) for an indefinite period or the concurrent use of a pre-erythrocytic stage vaccine or prophylactic therapy in children to protect those at risk from disease as immunity is lost in the population.

**Conclusions:**

Effective interventions will result in rapid decreases in clinical disease across all transmission settings while population-level immunity is maintained but may subsequently result in increases in clinical disease many years later as population-level immunity is lost. A dynamic, evolving intervention programme will therefore be necessary to secure substantial, stable reductions in malaria transmission.

## Introduction

Previous attempts at malaria eradication failed, in part, because of a lack of political will to sustain control efforts in the face of apparently diminishing returns [1]. A better understanding of the effect of reducing malaria transmission on the dynamics of infection and disease is essential if 21^st^ century attempts to eliminate malaria are to be more successful than those of the 20^th^ century. One of the most effective interventions currently recommended for reducing the burden of malaria infection and disease is the use of insecticide treated bed-nets (ITNs). ITNs reduce the number of anopholenes within a household by both repelling and killing mosquitoes and thus reduce the number of infectious bites received; ITNs thus reduce the incidence of infection and onward transmission [2]. Recently, a population-based analysis of the roll-out of ITNs across Kenya demonstrated that such interventions, if implemented with high coverage, could have a significant impact on the incidence of clinical disease at a national level [3], although it remains difficult to identify the impact of ITN in the general trends in decreasing malaria incidence now being reported across Africa [4], [5]. This has lead to a shift in guidance on interventions, with ITNs now promoted as the primary intervention across all transmission settings, and a key tool for those who consider eradication feasible [6]. In addition to ITNs, intermittent preventive therapy (IPT) is being widely studied as an intervention to reduce morbidity in infants (IPTi), children (IPTc) and pregnant women (IPTp). Several trials of IPTi have reported significant reductions in clinical disease [7]. Precisely how IPT protects against disease is unclear but is likely to be via a combination of clearance of existing blood stage infections and prophylaxis against reinfection [8] and will be affected by host immunity, levels of drug resistance and transmission dynamics [9], [10]. These interventions should work alongside improvements in diagnosis, clinical management and living conditions to reduce the morbidity and mortality associated with malaria infection [11].

Whilst there is no doubt that both ITNs and IPT will decrease the immediate risk of disease, concern remains that any exposure-reducing interventions, if implemented in highly endemic settings, could result in loss of (or failure to acquire) protective immunity and increase the overall burden of disease in the long term. Such concerns were highlighted by the observation that hospital admission rates for severe malaria amongst children are higher in endemic areas with moderate transmission than in endemic areas with high transmission due to the earlier development of clinical immunity in high transmission areas [12], [13]. Other studies have demonstrated a higher proportion of cases resulting in cerebral malaria and a higher case fatality rate in areas with lower transmission intensity, albeit with a lower overall malaria burden [14] although these results are not mirrored across all studies [15]. In studies of malaria chemoprophylaxis there is good evidence of a “rebound effect” after chemoprophylaxis is stopped [16], [17]. The likely consequences of sustained reductions in malaria transmission are thus very unclear [7].

Concerns about increasing incidence of disease following the introduction of an intervention are not unique to malaria. Indeed, over 20 years ago similar concerns arose over the introduction of a rubella vaccine in infants. Rubella infection is of most concern if acquired by pregnant women as it can give rise to congenital abnormalities in the unborn child, whereas in young children symptoms are generally mild. By reducing the overall force of infection in the population, it was predicted that disease would shift to older age-groups and hence the incidence of congenital rubella syndrome may increase if vaccine coverage was not sufficiently high [18]. More recently, Coleman et al. [19] noted that this effect, termed “endemic stability” in early veterinary research, would exist for any infectious disease in which two conditions were met–1) disease is more likely (or more severe) in older age-groups and 2) following an initial infection there is a reduced probability that future infections will occur or will result in disease.

In this paper we use a previously validated mathematical model to explore the transient and long-term population impact of exposure-reducing interventions for malaria [20]. This transmission model combines the development of two types of human immunity–one which reduces the development of clinical disease as individuals age and is dependent on past exposure and a second age-dependent physiological process which increases the rate at which parasites are cleared and is developed at a later age (10 years plus). These processes were combined into a full parasite transmission model. We use the model to explore the transient dynamics of malaria disease incidence following different interventions, including the use of ITNs and concurrent use of either IPT or a pre-erythrocytic stage vaccine, across a range of endemic settings.

## Methods

### Model Structure and Parameters

We use a modified version of the compartmental full transmission model described in Filipe et al. [20]. Briefly, the model stratifies the human population into 5 states–susceptible, exposed, clinical disease, asymptomatic infection and subpatent infection- and the mosquito population into 3 states–susceptible, exposed and infectious. Susceptible individuals can acquire new infections with a force of infection dependent on the level of infectiousness in the mosquito population. Super-infection can lead to disease in individuals who are currently asymptomatic. Clinical immunity develops over time dependent on the force of infection in the population and reduces the probability that an individual will develop clinical disease. Parasite immunity develops as individuals' age and reduces the amount of time spent in the asymptomatic patent infection state (mimicking a reduction in parasite density and hence onward infectiousness). Our previous model fitting suggests that the loss of both clinical and parasite immunity occurs over a period of years rather than weeks or months [20]. Although there are relatively little data on the duration of immunity, this is consistent with one study from Madagascar that reported reduced rates of fever in infected individuals born prior to the extensive successful malaria control program initiated in the 1950's, suggesting long-term immunological memory [21]. Full details of the model are given in Additional Information S1.

### Incorporation of interventions

We consider the impact of three interventions. Firstly, the use of ITNs is assumed to reduce the mosquito density per person in the community uniformly across all age-groups. Similar results are obtained if alternatively we reduce the biting rate. Secondly we consider the use of IPT in either infants or children aged up to 9 years of age. We assume that IPT would only be given to those who were not showing symptoms of disease (with those individuals showing clinical symptoms receiving treatment) and that clearance of parasite through IPT has 70% efficacy and is rapid (mean 1 day). 70% of those who have received IPT and cleared parasites are then fully protected from further infection for the prophylactic period (mean of 30 or 60 days). Finally we consider the use of a pre-erythrocytic stage vaccine which we assume provides partial protection against infection (with the extent of protection depending on vaccine efficacy–here we consider 30% [22], [23] or 90%) through reducing the force of infection experienced in those who are vaccinated. Individuals are vaccinated at a fixed rate set so that 50% of the susceptible population would be vaccinated after 5 years of a vaccination programme. We assume that those that are infected at the time of vaccination are also successfully treated. Following a period of time (mean of either 10 or 50 years) the vaccine wanes but these individuals do not receive any further vaccinations. For all three interventions we the development of immunity is not changed i.e. individuals continue to develop clinical immunity at rate depending on the force of infection in the community in which they live and parasite immunity at a rate dependent on their age.

### Model Validation

**Figure 1:**
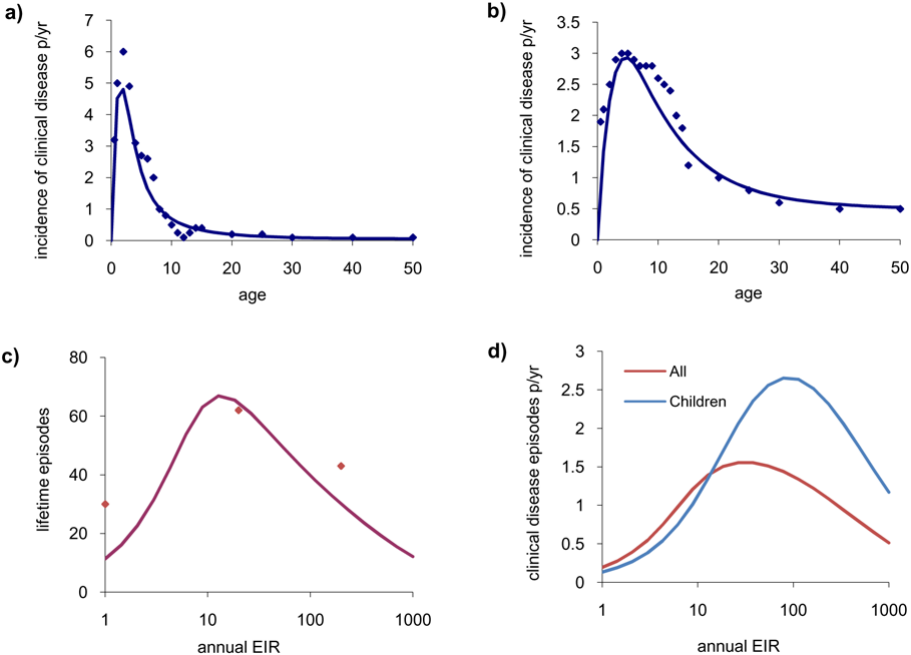
Comparison of model output with data on the incidence of clinical disease by age and lifetime number of episodes by EIR. The points are data and the lines denote model output. Model parameters are given in Additional Information S1 and are constant across runs except for EIR. a–b) Incidence of clinical disease by age measured in a) Dielmo and b) Ndiop in Senegal with EIR = 200 ibppy in Dielmo and 30 ibppy in Ndiop as reported in [24]. c) Lifetime number of episodes reported from Dielmo, Ndiop and Dakar in Senegal shown as data points by EIR [24]. The line shows the relationship between lifetime clinical episodes and EIR predicted by the model. d) Modelled relationship between EIR in endemic areas and expected clinical episodes per person (defined as symptomatic disease which results in treatment) across all ages (in red) and in children aged 0–9 years (in blue).

In our previous model validation we focused on identification of key parameters by matching to parasite prevalence patterns by age across the whole age spectrum and a variety of endemic settings [20]. In Figure 1 we show additional fits of the model to the incidence of clinical disease by age collected from a single comprehensive longitudinal study of malaria undertaken in two villages (Dielmo & Ndiop) in Senegal[24] (Figure 1a) plus lifetime clinical episodes of malaria from these two villages and Dakar (Figure 1b) [24].

## Results

### Endemic settings

Our model predicts that lifetime clinical disease episodes will peak in areas with moderate transmission (EIRs in the region 20–40 infectious bites per person years (ibppy) for the parameter values assumed) and decrease at higher EIRs due to the exposure-driven development of immunity. As a consequence, at any point in time, the incidence of clinical disease is also predicted to peak in moderate transmission settings (Figure 1d).

**Figure 2:**
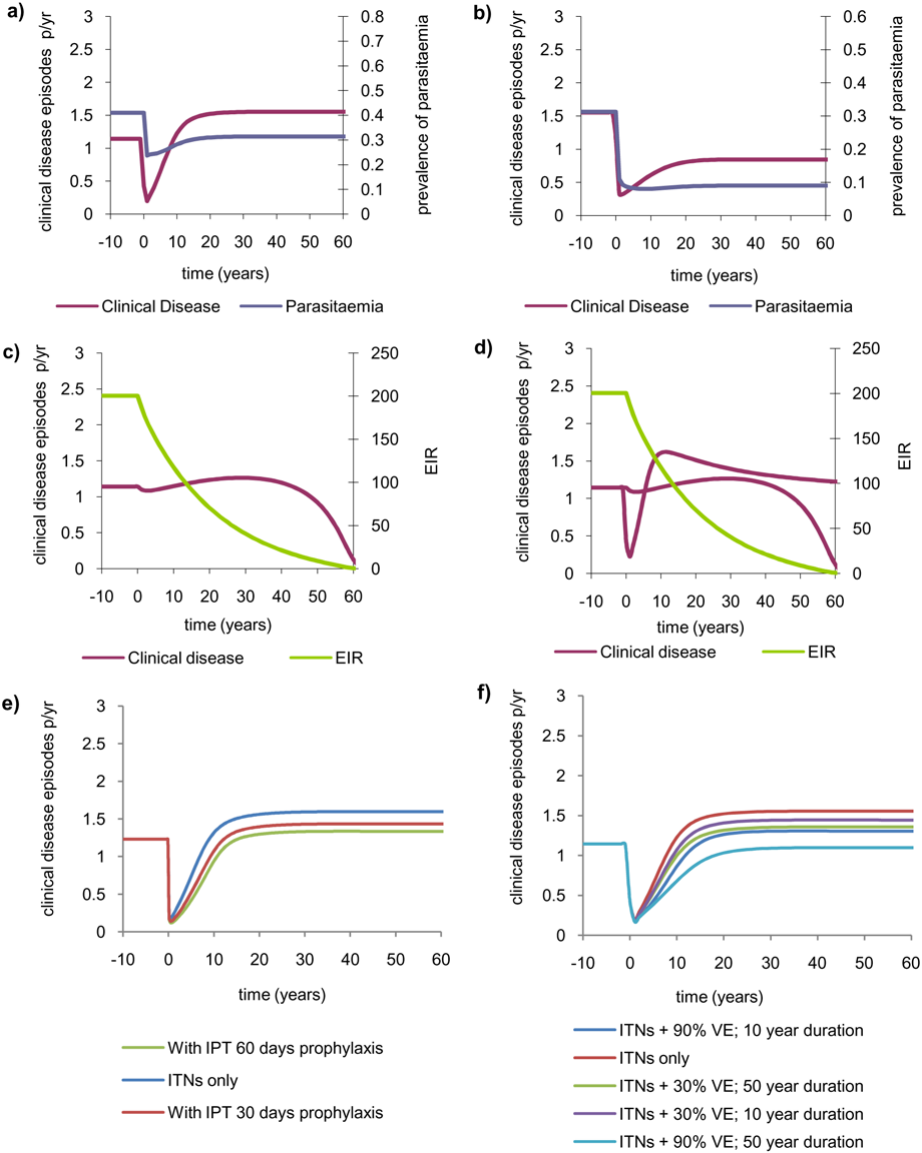
The long-term impact of sustained exposure-reducing interventions (represented by a decrease in density of female anophelines). Results show the impact on the incidence of clinical disease (shown in red on the left axis for figures a–d), the prevalence of parasitaemia (shown in blue on the right axis for figures a–b) and consequent changes in the EIR (shown in green on the right axis for figures c–d). Prior to the intervention the disease is endemic and interventions are introduced at year 0. a) Use of ITNs which lead to a continuous (but rapid) reduction in transmission intensity from EIR = 200 ibppy to EIR = 30 ibppy. b) Use of ITNs which reduce the transmission intensity from EIR = 30 ibppy to EIR = 5 ibppy. c) Impact of a continually improving intervention. We assume that ITNs are introduced in a setting with EIR = 200 ibppy and lead to a 5% annual reduction in the EIR shown in green. d) Impact of an intervention that gradually wanes starting with EIR = 200. We assume that the reduction in mosquito density takes the form 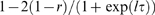 where *r* is the initial reduction, 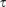
is the time since the intervention and *l* = 1e-3 is a parameter determining the speed of loss of effectiveness. The effect of this waning on EIR is shown in green. e) As in a) but additionally assuming that IPTi is given at 2, 3 and 10 months of age and IPTc is given 6-monthly to children aged from 24 months to 9 years. Two scenarios for the duration of prophylaxis are considered–30 days and 60 days. f) As in a) but with additional use of a pre-erythrocytic stage vaccine administered to the whole population which is assumed to reduce the susceptibility to infection by either 30% (to mirror Phase II results from the RTS,S/A202A vaccine although these results refer to morbidity rather than infection) [22], [23] or 90% (an optimistic value). Two scenarios for the duration of protection are considered–long-lived (mean 50 years) or short-lived (mean 10 years).

If exposure is reduced at a population level, for example through the widespread use of ITNs, this will impact on transmission, reducing the EIR. Depending on the EIR in the setting prior to the intervention and the overall effectiveness of the intervention in reducing transmission, there may be a negative (Figure 2a) or positive (Figure 2b) impact on clinical disease in the long-term. More generally, if the EIR in a setting is relatively high then an intervention will need to be highly successful to ensure that no increase in clinical disease occurs, whilst if the EIR is in the range in which a peak in clinical disease incidence is predicted (approximately 30 ibppy in this model) then even a moderately effective intervention will reduce clinical disease incidence. These results mirror those found more generally for the range of infectious diseases in which endemic stability is predicted to occur as well as in simpler models for malaria immunity [18], [19], [25]–[27].

This endemic pattern is driven in our model by the acquisition and loss of clinical immunity which is primarily determined by the force of infection in the population. Thus the acquisition and loss of immunity alone can result in a pattern of “endemic stability” as defined by Coleman et al. [19] without additional need to invoke an increase in the severity of disease with age.

### Transient dynamics and adverse effects of changing incidence

The success of an intervention is typically measured by the patterns of disease incidence in the 1–2 years following the intervention. In Figure 2a–b we show that the population transmission dynamics predict a transient drop in clinical disease incidence as transmission intensity is reduced in a fully immune population. However, we also show that, because immunity at a population level develops (or is lost) over a period of decades, this drop in disease level is followed by a steady rise (as immunity is gradually lost) to a new, endemic equilibrium which is not reached for decades (Figure 2a–b) and which can be either higher or lower than that prior to interventions depending on transmission levels before and after the intervention.

These epidemiological predictions should, however, be interpreted with caution as they assume that the effectiveness of the intervention is sustained for many decades whereas in practice long-term effectiveness will be affected by developing drug and insecticide resistance, socio-demographic changes, and changes in control and treatment policies. However, if intervention effectiveness can not only be sustained but also improved over time, the adverse outcome predicted in Figure 2a can be averted. For example, Figure 2c shows a scenario in which exposure is reduced gradually (by 5% per year rather than rapidly as in Figure 2a) which may represent increasing coverage of ITNs or additional interventions being implemented–for example regular indoor residual spraying (IRS). In this scenario the reduction in EIR is initially insufficient and results in an increase in clinical disease, but after a longer period of time (>40 years) the EIR has declined sufficiently to reduce clinical disease incidence below the levels seen prior to introduction of ITNs. In this simple scenario, the drop in clinical incidence continues until local elimination occurs 60 years after the start of the intervention. This outcome is unlikely in practice as it relies on the assumption that the intervention is sustained and conditions are undisturbed for 60 years. However, it also demonstrates that even local elimination would require many decades of sustained and effective control effort.

If the effectiveness of the intervention gradually wanes, as shown in Figure 2d, the impact on immunity is likely to be minimal and the incidence of clinical disease will return to pre-intervention settings. However, unexpected temporal patterns in disease incidence may occur. For example, if the intervention is introduced in a high transmission setting and is initially very successful we may observe an initial transient drop in clinical disease incidence, followed some 10 years later by a rise towards a peak in incidence higher than prior to the intervention, before clinical incidence returns to pre-intervention levels (Figure 2d). This ‘zig-zag’ behaviour results from the trade-off between a steady rebound in EIR and a loss followed by a regain of protective immunity at a population level. A naive interpretation of such a transient pattern would be that the intervention is initially very successful (drop in incidence), then begins to fail (as incidence increases) and in a third phase improves as incidence decreases. During the second and third stages of this process, the naïve interpretation may be incorrect (or at least incomplete) and may lead to erroneous decision making.

### Combining interventions to reduce the chance of adverse outcomes

An alternative option to prevent any adverse outcome of an exposure-reducing intervention would be to incorporate multiple interventions simultaneously. Figure 2e shows the potential impact of the same exposure-reducing intervention as Figure 2a, but this time supplemented with extensive IPTi and IPTc. IPT in infants (age<1 year) provides little additional protection since as the EIR is reduced through ITNs the burden of disease is predicted to shift to young children (aged 2–5 years). However, a comprehensive IPT intervention in children (6-monthly treatment of children aged 2–9 years with an efficacious long-acting drug such as mefloquine or piperaquine over a 30-day prophylactic period) can significantly reverse the adverse outcome of other interventions. Furthermore, if a drug with a longer prophylactic period (in the example here 60 days) or more-frequent treatment is used then the adverse outcome initially predicted could be completely prevented.

Vaccination could also be combined with exposure-reducing interventions. Figure 2f shows the impact of a pre-erythrocytic vaccine which, for simplicity, we assume reduces the risk of infection but does not affect the development of clinical immunity. Under the characteristics of the current vaccination under consideration (RTS,S/A202A–which provides ∼30% efficacy against malarial morbidity [22], [23]) the adverse outcome can be reduced but not completely reversed, even if the duration of protection provided by the vaccine is very long. However, a vaccine with substantially higher efficacy (e.g. one with 90% efficacy) could be beneficial and prevent the adverse outcome if the duration of protection is sufficiently long (or if the duration is extended with a boosting programme). Thus it is clear that by combining interventions that act in different ways the adverse outcomes predicted by our model could be substantially or almost completely negated.

### Monitoring for increases in the incidence of clinical disease

**Figure 3:**
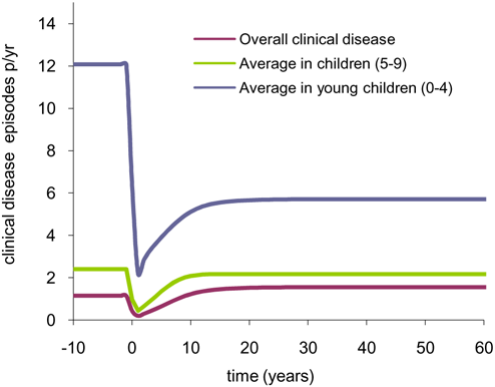
Impact of a sustained ITN intervention on disease incidence in infants and young children. A reduction in EIR from 200 to 30 ibppy is assumed (as in Figure 2a).

Given the many uncertainties in malaria transmission dynamics and limitations in its monitoring, it will be difficult to assess which if any of the scenarios outlined above may occur once interventions are introduced. Thus it is of interest to identify a group of individuals in whom any adverse results would be detected early. Figure 3 shows that a larger relative increase in incidence of clinical disease compared to pre-intervention levels will be observed in children aged 5–9 years (who will fail to acquire any protective immunity) and hence this may be the best group to monitor for rebound effects in the early years of a control programme. Infants and younger children will continue to benefit from protection conferred by maternal immunity (which depends on transplacental transfer of antimalarial antibodies and is likely to persist in the mother for some years in the absence of ongoing infection [28]) and so will experience a greater drop in clinical incidence until their mothers' immunity has waned, after which more rapid rises in clinical disease are predicted.

## Discussion

Our results demonstrate that the higher incidence of clinical malaria that has been observed in moderate transmission settings relative to high transmission settings [24] may be a result of “endemic stability” driven by the rapid development of clinical immunity in high transmission settings. This mechanism alone appears to reproduce the patterns of clinical disease by transmission setting without additionally incorporating a mechanism by which the severity of disease increases with age (as postulated in Coleman *et al.* [19]). We believe that our model for the acquisition and loss of immunity adequately mimics the biology of the infection and its potential impact on all clinical disease. However, this does not exclude other factors being involved in producing this pattern, such as purely physiological effects which are likely to determine the spectrum of disease at different ages.

Our model gives important new insights into the expected dynamics of malarial disease incidence following an intervention. Whilst, given the absence of detailed longitudinal data, it is impossible to precisely define the timescale of the transient impact of any intervention, our results suggest that these timescales will be in the order of many years to decades. Furthermore, our examples illustrate that in the years following an intervention we may initially see a temporary decline in incidence (as a result of a decline in transmission intensity in a highly immune population) whereas after this time disease incidence could, in the worst scenarios, increase to higher levels than prior to the intervention. It is important to emphasise that this eventual increase in disease incidence does not necessarily indicate any loss of effectiveness of the intervention. Such adverse scenarios are characteristics of settings in which endemic stability dominates, and indeed similar transient dynamics were predicted for the impact of rubella vaccination on congenital rubella syndrome in developing countries and for chemotherapy and vaccination against helminth infections [18], [26]. Furthermore, the initial rapid declines in incidence predicted by our model appear consistent with those observed in studies of the introduction of ITNs reported to date [2]. However, our results also demonstrate that such adverse scenarios can be avoided by combining transmission-reducing interventions with either a partially-effective vaccine or prophylactic therapy, and ensuring that the effectiveness of the interventions is sustained over many years. To ensure that this goal is achieved, both the intervention itself and monitoring for any negative effects must remain in place for many years, possibly decades.

One important aspect not addressed here is the relationship between overall clinical disease and severe disease and childhood mortality. Because of the complex disease spectrum and the relationship between severe disease and age, the basis of which is not yet fully understood, we chose not to explicitly incorporate this directly in our model. However, there is substantial evidence demonstrating that severe malarial anaemia peaks in infants under 2 years of age in high transmission settings whereas cerebral malaria is more common in children over 5 years of age in lower transmission settings [14]. Hence any shift in disease incidence into older age-groups is also likely to give rise to shift in clinical presentation of severe malaria disease.

Clearly there are limitations to any modelling exercise. Although we were able to validate the static aspects of our model over the whole age range of populations across a wide variety of endemic settings, there are limited data from longitudinal studies with which to validate the dynamics. Secondly, whilst one may argue that some aspects of malaria epidemiology are not well-captured within our proposed compartmental framework, we believe that it captures the main features of malaria epidemiology that will impact at a qualitative level on the dynamics of disease incidence following an intervention. Finally, in our scenarios we assume that each individual is protected by both the exposure-reducing interventions and the prophylactic therapy or vaccination that they receive, but continue through this period to develop clinical immunity at a rate dependent on the overall level of transmission in the community in which they live. There is some evidence that the development of acquired immunity to malaria is enhanced in partially-protected individuals, particularly under high transmission [9], [29]. Such a scenario would reduce the chance of adverse outcomes as transmission is reduced from high to moderate levels.

How then should we assess the impact of future exposure-reducing interventions? Firstly, a clearer understanding of the development of acquired immunity is critical to these hypotheses and hence interpretation of all intervention studies. The predictions of our two-stage immunity model depend on the degree of exposure that is required to develop immunity and the timescales over which immunity is gained and lost. In this work we assume that clinical immunity is developed at a rate proportional to the EIR in each setting and has a half-life of approximately 7 years, and that parasite-clearance immunity has a half-life of approximately 14 years. The former parameter is particularly important in the predictions made here (see Additional Information S1). These parameters reproduce epidemiological patterns [20] and are consistent with epidemiological observation [21] but have not been formally measured in any malaria endemic setting. Improving our understanding of immunity mechanisms requires long-term monitoring of immunological markers and clinical disease episodes in cohorts of individuals in different malaria endemic settings. Secondly, the model highlights the need for detailed assessment of the current endemic levels of disease prior to interventions being scaled up. Recent research undertaken as part of the Malaria Atlas Project has gone some way to achieve this and suggests that high EIR settings may be less common than previously believed [30]. If this is the case then the adverse scenarios that we describe may occur infrequently. Thirdly, whilst studies have demonstrated a significant reduction in mortality or clinical disease in the months or years after a successful intervention, longer-term follow-up of protected communities is important in order to monitor subsequent impact and to differentiate it from reductions in transmission through e.g. changing climate and social improvements. Finally, the shape of the long-term relationship between exposure (EIR) and clinical disease in our model demonstrates that, if multiple effective interventions are implemented, concurrently, with high coverage, it is possible to offset the negative impact of reducing immunity through driving transmission to low levels [14]. In fact, as EIR falls and there are fewer immune individuals, the proportion of asymptomatic infections will also fall and thus a greater proportion of infected people will seek antimalarial treatment. Under these conditions, prompt treatment of cases with a highly effective antimalarial regimen can contribute significantly to transmission reduction [31], [32]. Several countries in sub-Saharan Africa have free distribution of long-lasting ITNs supplemented by rounds of insecticide-residual spraying and have changed first-line therapy to artemisinin-based combinations. Sustaining both control interventions and effective case management for many years, possibly decades, should remain the primary goal of all intervention programmes and it is essential that these long-term goals are matched with financial commitments.

## Supporting Information

Additional Information S1 Mathematical details of the model and parameter sensitivity analysis
(0.24 MB DOC)
