# Supplementary material for: Loss of Population Levels of Immunity to Malaria as a Result of Exposure-Reducing Interventions: Consequences for Interpretation of Disease Trends
Source: PLoS One. 2009 Feb 9;4(2):e4383. doi: 10.1371/journal.pone.0004383 (PMC2634959; doi:10.1371/journal.pone.0004383)
Supplement: Additional Information S1 — Mathematical details of the model and parameter sensitivity analysis (0.24 MB DOC) [file pone.0004383.s001.doc]

Loss of population levels of immunity to malaria as a result of exposure-reducing interventions: Consequences for interpretation of disease trends

Additional Information

Ghani, Azra C^1,2^; Sutherland, Colin J^3^; Riley, Eleanor M^3^; Drakeley, Chris J^3^; Griffin, Jamie T^1^; Gosling, Roly D^3^; Filipe, Joao A N^2,4^

1. MRC Centre for Outbreak Analysis & Modelling, Department of Infectious Disease Epidemiology, Imperial College London, London, UK

2. Department of Epidemiology & Population Health, London School of Hygiene &Tropical Medicine

3. Department of Infectious and Tropical Diseases, London School of Hygiene & Tropical Medicine, London, UK

4. Department of Plant Sciences, University of Cambridge, Cambridge, UK

**Methods**

**Mathematical model**

We used a slightly extended version of a previously published compartmental transmission model which tracks the transmission of malaria parasites between human and mosquito populations [1]. We present here details of the model for clarity of notation so that in later sections in which we introduce the interventions the same notation can be used. In brief, the age-structured model stratifies the human population into susceptible or non-infected state S_H_, exposed but not yet infectious E_H_, infected with symptomatic disease (which includes severe disease as well as other clinical cases that would be identified and treated) D_H_, asymptomatic patent infection A_H_ and infected with sub-patent parasite density U_H_. The mosquito population is either susceptible S_M_, latent E_M_ or infectious I_M_ with the dynamics for this population as in the Ross-Macdonald model. The equations for the human population are:

where $\Lambda(\alpha)$is the force of infection experienced by a person of age α, 1/*h* is the mean latent period, φ is the clinical susceptibility or proportion that develop symptomatic disease (which depends on their immune status – see below), *f* is the proportion of symptomatic cases who receive effective drug treatment and recover at rate r_T_, and r_D_ is the natural recovery rate for those who do not receive treatment . r_A_ is the rate at which asymptomatic infections become subpatent and r_U_ is the rate at which subpatent infections are cleared. If clinical treatment or natural recovery successfully removes parasites (with probability φ which depends on the host’s immune status – see below) the host returns to the susceptible state; otherwise they move to the asymptomatic state. Those in the asymptomatic state may additionally develop disease through superinfection at rate φΛ(α). We assume the population size remains constant and for simplicity ignore extra mortality due to disease because the proportion of fatal cases is comparatively small.

The equations for the mosquito population are:

$$\begin{matrix} \frac{dS_{M}}{dt}=-\Lambda_{M} S_{M}+\mu-\mu S_{M} \\ \frac{dE_{M}}{dt}=\Lambda_{M} S_{M}-\Lambda_{M}(t-\tau) S_{M}(t-\tau)e^{-\mu\tau}-\mu E_{M} \\ \frac{dI_{M}}{dt}=\Lambda_{M}(t-\tau) S_{M}(t-\tau)e^{-\mu\tau}-\mu I_{M} \end{matrix}$$

where μ is the mosquito mortality rate, τ is the parasite extrinsic latent period, and exp(-μτ) is the probability that the mosquito survives the extrinsic latent period. We assume that the mosquito population is at equilibrium in relation to the human population dynamics so that the force of infection experienced by mosquitoes is:

$$\Lambda_{M}=a\int_{0}^{\alpha\max} \rho(\alpha)\left[c_{D}D(\alpha-\delta)+c_{A}A+c_{U}U\right]d\alpha$$

where *c_i_ (i=D,A,U)* is the human state-specific infectivity (probability of transmitting gametocytes to a biting mosquito), δ is the delay in clearance of gametocytes from peripheral blood circulation upon clearance of blood stages, and *ρ(α)* is the density of people of age α.

The maximum force of infection acting on humans is then given by:

$$\Lambda_{H}=bEIR =bm a I_{M}$$

where *EIR* denotes the entomological inoculation rate, *b* is the probability of inoculation of a human upon a mosquito infectious bite, *m* is the density of mosquitoes per human, *a* the mosquito biting rate on humans and I_M_ the prevalence of infectious mosquitoes. The age-dependent force of infection acting on humans is given by

$$\Lambda(\alpha)=\Lambda_{H}\text{(1-e}^{\text{-}\alpha/\alpha_{0}})$$

where α_0_ is a parameter. This function allows the model to incorporate an increase in exposure with age.

Two types of human immunity are incorporated dynamically in the model – clinical immunity and parasite immunity. We previously showed that these functions are those most consistent with data on parasite prevalence by age[1]. Clinical immunity, which reduces the probability φ that an infection results in clinical disease, has a value at birth conferred by the level of immunity in mothers at the time of birth (assumed to be half that in adults of child-bearing age taken as 25 years) and decays exponentially with mean *d_m_*. This is described by the partial differential equation:

$$\begin{matrix} \frac{\partial I_{m}}{\partial\alpha}+\frac{\partial I_{m}}{\partial t}=-\frac{I_{m}}{d_{m}} \\ I_{m}(0,t)=0.5I_{s}(25,t) \end{matrix}$$

Clinical immunity then accumulates with exposure at a rate determined by the force of infection and decays exponentially with mean *d_c_*. This process is described by:

$$\begin{matrix} \frac{\partial I_{c}}{\partial\alpha}+\frac{\partial I_{c}}{\partial t}=\Lambda(\alpha)-\frac{I_{c}}{d_{c}}, \\ I_{c}(0,t)=0. \end{matrix}$$

The overall level of clinical immunity is given by:

$$I_{S}(\alpha,t)=I_{M}(\alpha,t)+I_{C}(\alpha,t).$$

The susceptibility to developing clinical disease is assumed to be a nonlinear decreasing function of the clinical immunity level:

$$\varphi(I_{s})= \frac{1}{1+(I_{s}/H_{s})^{2}}.$$

Parasite immunity, which increases the rate at which parasites are cleared, is assumed to accumulate with age only (provided there is exposure) with a delay *d_l_* representing physiological development and decays exponentially with mean *d_a_*. The level of parasite immunity I_A_ is thus given by

$$\begin{matrix} \frac{\partial J_{A}}{\partial\alpha}=F\text{(1-e}^{\text{-}\alpha/\alpha_{0}})-\frac{J_{A}}{d_{l}} \\ \frac{\partial I_{A}}{\partial\alpha}=\frac{J_{A}}{d_{l}}-\frac{I_{A}}{d_{a}} \\ I_{A}(0)=J_{A}(0)=0 \end{matrix}$$

where F is a constant determining the rate of acquisition of parasite immunity and J_A_ represents a delay phase.

The natural recovery rate from clinical disease and from asymptomatic infection is therefore assumed to be a saturating increasing function of the immunity level I_A_:

$$r_{D}(I_{A})=r_{A}(I_{A})=r_{0}\left[1+(w_{A}-1)\frac{I_{A}^{2}}{H_{A}^{2}+I_{A}^{2}}\right]$$

where w_A_ is a parameter determining the maximum value that the recovery rate can take.

Tables S1 summarises the parameters in the model and their assumed values (some of which are modified slightly compared to those presented in[1]).

**Table S1 – Model Parameters**

| Parameter | Description | Estimates from literature | Value in model |
| --- | --- | --- | --- |
| *EIR* | Entomological Inoculation Rate – derived from other parameters | - | 200, 30, 5 |
| *α_0_* | parameter determining the rate at which exposure increases with age | Assumed value gives 95% of maximum exposure occurring by age 10 years | 3 years |
| *b* | probability that a human bitten by an infectious mosquito is infected | 0.3-0.5[2]; 0.03-0.13[3]; 0.1[4] | 0.25 |
| *r_T_* | rate of recovery from clinical malaria following treatment | Varies by drug: ACT 10 days, non-ACT up to 40 days; set to include time to clear gametocytes | 1/21day^-1^ |
|  |  | Parasitaemia detectable by microscopy:  211 days (95% CI: 155-268) [5]  Genotyping:  210 days (GeneScan), 152 days (RFLP) [6] |  |
| *r_0_* | rate of recovery from clinical malaria or asymptomatic to sub-patent (baseline value) |  | 1/180 day^-1^ |
| *r_U_* | rate of clearance of sub-patent infections | - | 1/180 day ^-1^ |
| *1/h* | mean latent period in humans | sporozoite inoculation to parasitaemia: 7-13 days [7]  patent parasitaemia to infectious gametocytes: 10-14 days [8]; 10 days [9] | 15 days |
| *c_D_, c_A_, c_U_* | probability of transmission from humans in states D, A and U respectively to mosquitoes for a single bite | 0.034-0.167 amongst gametocyte positives [10-12]  0.078-0.354 in general population samples [10,13-15] | 0.2, 0.05, 0.01 |
| $\tau$ | gametocyte clearance delay upon chemotherapy | Many studies show the presence of gametocytes up to 21 days –mean duration depends on drug | 21 days |
| *f* | proportion of symptomatic cases treated effectively | Varies widely | 0.5 |
| *µ_0_* | human natural mortality rate (assumed to be constant with age) | Derived from data in [16] | 0.05yr^-1^ |
| *ρ(α)* | density of people of age α | Derived from data in [16] |  |
| *αmax* | maximum age in the human population | - | 60 years |
| *a* | biting rate on humans by a female mosquito | 0.02-0.465 day-1 – [17] and references therein | 0.33 day ^-1^ |
| *µ* | mosquito natural mortality rate | 9-10 days at 28^0^C[18] | 0.10 day ^-1^ |
| *d_m_* | Mean duration of maternal protection | Severe infection: half-life 3-9 months[19] | 3 months |
| *d_s_* | Mean duration of clinical immunity | 5 years in [1] Higher value needed to reproduce lifetime episodes in [20] | 10 years  (half-life~6.9 years) |
| *d_l_* | latent period in the development of parasite immunity | As in [1] | 10 years  (half-life~6.9 years) |
| *d_a_* | Mean duration of parasite immunity | As in [1] | 20 years  (half-life~13.9 years) |
| *w_A_* | maximum amplification of baseline recovery rate | Assumes at maximum immunity parasite recovery rate has mean 1/6 days^-1^ | 30 |
| *H_S_* | level of clinical immunity at half saturation | As in [1] | 40 |
| *H_A_* | level of parasite immunity at half saturation | As in [1] | 1000 |
| *F* | Constant exposure determining rate of acquisition of parasite immunity | Adjusted to match parasite prevalence data (as in [1]) | 30 |

**Interventions**

We examined three different interventions in the model – a reduction in exposure (for example through the use of ITNs), the use of intermittent preventive therapy in infants (IPTi) , and the use of a pre-erythrocytic stage vaccine.

*a) Reduction in exposure*

A reduction in exposure and thus in EIR was assumed to take place via a reduction in the mosquito density and this reduction was considered to be the same across all age-groups. Similar results are obtained if alternatively the mosquito biting rate *a* is reduced. We considered two scenarios: a reduction in overall EIR from 200 to 30 infectious bites per person year (ibppy) and a reduction in EIR from 30 to 5 ibbpy. The intervention was introduced once the model had reached endemic equilibrium (for both prevalence and human immunity stages) and was assumed to occur immediately. We also considered a scenario in which EIR was reduced gradually (Figure 3d) and one in which the effectiveness of the intervention waned over time (Figure 3e-f).

b) *Intermittent Preventive Therapy in infants (IPTi)and children (IPTc)*

Intermittent preventive therapy (IPT) was assumed to be given only to those who were not showing signs of clinical disease (we assumed that these individuals would receive treatment). IPT therefore clears infection in those with parasiteamia (A_H_) and subpatent infection (U_H_). We assume that clearance of parasites has efficacy of 70% and if successful is rapid (mean duration to clearance of 1 day). IPT is then assumed to provide complete protection from infection to 70% of those who receive it (protecting also those who receive it while in the susceptible state S_H_) for a mean duration of either 30 or 60 days. In the results shown IPTi was given at 2, 3 and 10 months, a pattern consistent with the trials undertaken to date [21]. For IPTc we considered a comprehensive schedule in which children from 24 months to 9 years of age were given IPT at 6-month intervals. The equations for the human infection process were therefore modified as shown below:

$$\begin{matrix} \frac{\partial S_{H}}{\partial t}+\frac{\partial S_{H}}{\partial\alpha}=-\Lambda(\alpha) S_{H}+\varphi R_{D} D_{H}+r_{U}U_{H}-\gamma\varepsilon(\alpha)S_{H}+\eta I_{H} \\ \frac{\partial E_{H}}{\partial t}+\frac{\partial E_{H}}{\partial\alpha}=\Lambda(\alpha) S_{H}-h E_{H}-\beta\gamma\varepsilon(\alpha)E_{H} \\ \frac{\partial D_{H}}{\partial t}+\frac{\partial D_{H}}{\partial\alpha}=\varphi h E_{H}+\varphi\Lambda(\alpha) A_{H}-R_{D} D_{H} \\ \frac{\partial A_{H}}{\partial t}+\frac{\partial A_{H}}{\partial\alpha}= (1-\varphi)h E_{H}+(1-\varphi) R_{D} D_{H}-\varphi\Lambda(\alpha) A_{H}-r_{A}A_{H}-\beta\gamma\varepsilon(\alpha)A_{H} \\ \frac{\partial U_{H}}{\partial t}+\frac{\partial U_{H}}{\partial\alpha}=r_{A}A_{H}-r_{U}U_{H}-\beta\gamma\varepsilon(\alpha)U_{H} \\ \frac{\partial I_{H}}{\partial t}+\frac{\partial I_{H}}{\partial\alpha}=\gamma\varepsilon(\alpha)\left(S_{H}+\beta E_{H}+\beta A_{H}+\beta U_{H}\right)-\eta I_{H} \end{matrix}$$

where I_H_ is the prophylactic state for those who have received IPT, ε(α) is the age-dependent rate of receiving IPT, β=0.7 is the imperfect clearance of parasites, γ=0.7 represents imperfect protection from further infection and 1/η is the mean duration of protection against infection conferred by IPT.

c) *Use of a pre-erythrocytic stage vaccine*

We assume that a pre-erythrocytic stage vaccine will reduce but not eliminate the risk of infection and thus that vaccine efficacy reduces the susceptibility to infection (*b*) of vaccinated individuals by a factor $\theta$. Furthermore, we assume for simplicity that being vaccinated does not directly affect an individual’s development of immunity and hence that they continue to develop immunity at a rate dependent on the transmission intensity in the community in which they live. Individuals are assumed to be vaccinated at a rate $\xi$and we assume that the efficacy of the vaccine wanes at a rate $\gamma$. At vaccination we assume that any infection is also treated. Once the vaccine has waned individuals become as susceptible as non-vaccinated individuals of the same age but are tracked in a separate compartment as we assume that re-vaccination does not occur. Thus the equations for the human infection process become:

where the subscripts V and W denote vaccinated and previously vaccinated but no longer effective (waned) respectively.

**Numerical Solutions**

The partial differential equations were approximated with ageing occurring exponentially. For endemic solutions (prior to interventions) exact solutions for the immunity functions were calculated (see Supplementary Information in [1]) and the transmission model then solved numerically using yearly age-groups. For the runs with interventions the clinical immunity equations were solved numerically using monthly age-groups to capture the rapid development of clinical immunity at high EIR. Fourth-order Runge-Kutta routines were used for numerical solutions in Berkeley Madonna software with a time-step of 1 day.

**Sensitivity Analyses**

The results presented in the main paper are particularly sensitive to the mean duration of clinical immunity which determines susceptibility to disease. However, this is confounded with the rate of acquisition of immunity and hence cannot be directly determined without longitudinal data. Here we present a sensitivity analysis for these parameters to demonstrate the impact that they have on i) the pattern of endemic disease at different EIR predicted by the model and ii) the impact of an intervention that reduces the EIR from 200 to 30 (as in Figure 2a).

The mean duration of clinical immunity influences the shape and peak of the relationship between EIR and clinical disease (Figure S1). If clinical immunity is shorter-lived then the relationship is steeper and the peak in clinical incidence occurs at slightly higher EIRs.


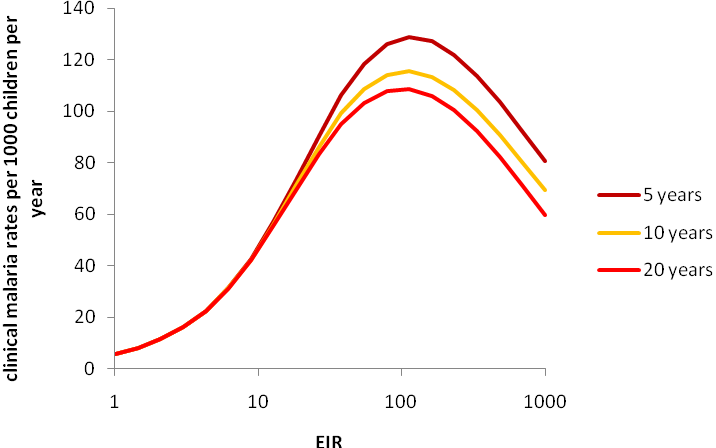


**Figure S1 – Relationship between EIR and the incidence of clinical disease in children up to 9 years of age for different values of the mean duration of clinical immunity d_S_.**

Figure S2 shows the impact that this parameter has on the timescales of the intervention. We focus here on the scenario shown in the main text Figure 2a, in which EIR is reduced from 200 ibppy to 30 ibppy. If the duration of clinical immunity is shorter than assumed we expect a higher baseline rate of clinical disease for a given EIR. Therefore, following the intervention the increase in clinical cases occurring through loss of immunity at a population level will be faster and will reach a higher peak than in the baseline scenario. For durations of clinical immunity longer than assumed, the reverse is true. However, in all cases the final new endemic equilibrium will not be reached for at least a decade.

**Figure S2 – Pattern of change in clinical disease incidence for different values of the mean duration of clinical immunity d_S_.**

Detailed sensitivity analyses for other parameters determining the patterns of age-prevalence of parasitaemia are presented in Filipe et al.[1]

**References**

1. Filipe JAN, Riley EM, Drakeley CJ, Sutherland CJ, Ghani AC (2007) Determination of the mechanisms driving the acquisition of immunity to malaria using a mathematical transmission model. PLoS Computational Biology 3: e255.

2. Rickman LS, Jones TR, Long GW, Paparello S, Schneider I, et al. (1990) Plasmodium falciparum-infected Anopheles stephensi inconsistently transmit malaria to humans. Am J Trop Med Hyg 43: 441-445.

3. Beier JC, Oster CN, Onyango FK, Bales JD, Sherwood JA, et al. (1994) Plasmodium falciparum incidence relative to entomologic inoculation rates at a site proposed for testing malaria vaccines in western Kenya. Am J Trop Med Hyg 50: 529-536.

4. Dietz K, Molineaux L, Thomas A (1974) A malaria model tested in the African savannah. Bull World Health Organ 50: 347-357.

5. Sama W, Dietz K, Smith T (2006) Distribution of survival times of deliberate Plasmodium falciparum infections in tertiary syphilis patients. Trans R Soc Trop Med Hyg 100: 811-816.

6. Falk N, Maire N, Sama W, Owusu-Agyei S, Smith T, et al. (2006) Comparison of PCR-RFLP and Genescan-based genotyping for analyzing infection dynamics of Plasmodium falciparum. Am J Trop Med Hyg 74: 944-950.

7. Eyles DE, Young MD (1951) The duration of untreated or inadequately treated Plasmodium falciparum infections in the human host. J Natl Malar Soc 10: 327-336.

8. Nedelman J (1989) Gametocytaemia and infectiousness in falciparum malaria: observations and models. Adv Dis Vector Res 6: 59-89.

9. Jeffery GM, Eyles DE (1955) Infectivity to mosquitoes of Plasmodium falciparum as related to gametocyte density and duration of infection. Am J Trop Med Hyg 4: 781-789.

10. Bonnet S, Gouagna LC, Paul RE, Safeukui I, Meunier JY, et al. (2003) Estimation of malaria transmission from humans to mosquitoes in two neighbouring villages in south Cameroon: evaluation and comparison of several indices. Trans R Soc Trop Med Hyg 97: 53-59.

11. Graves PM, Burkot TR, Carter R, Cattani JA, Lagog M, et al. (1988) Measurement of malarial infectivity of human populations to mosquitoes in the Madang area, Papua, New Guinea. Parasitology 96 (Pt 2): 251-263.

12. Coleman RE, Kumpitak C, Ponlawat A, Maneechai N, Phunkitchar V, et al. (2004) Infectivity of asymptomatic Plasmodium-infected human populations to Anopheles dirus mosquitoes in western Thailand. J Med Entomol 41: 201-208.

13. Boudin C, Olivier M, Molez JF, Chiron JP, Ambroise-Thomas P (1993) High human malarial infectivity to laboratory-bred Anopheles gambiae in a village in Burkina Faso. Am J Trop Med Hyg 48: 700-706.

14. Muirhead-Thomson RC (1957) The malarial infectivity of an African village population to mosquitoes (Anopheles gambiae); a random xenodiagnostic survey. Am J Trop Med Hyg 6: 971-979.

15. Githeko AK, Brandling-Bennett AD, Beier M, Atieli F, Owaga M, et al. (1992) The reservoir of Plasmodium falciparum malaria in a holoendemic area of western Kenya. Trans R Soc Trop Med Hyg 86: 355-358.

16. Drakeley CJ, Carneiro I, Reyburn H, Malima R, Lusingu JPA, et al. (2005) Altitude-dependent and -independent variations in Plasmodium falciparum prevalence in northeastern Tanzania. Journal of Infectious Diseases 191: 1589-1598.

17. Gu W, Mbogo CM, Githure JI, Regens JL, Killeen GF, et al. (2003) Low recovery rates stabilize malaria endemicity in areas of low transmission in coastal Kenya. Acta Trop 86: 71-81.

18. Macdonald G (1957) The epidemiology and control of malaria. London: Oxford University Press.

19. Gupta S, Snow RW, Donnelly CA, Marsh K, Newbold C (1999) Immunity to non-cerebral severe malaria is acquired after one or two infections. Nat Med 5: 340-343.

20. Trape JF, Rogier C (1996) Combating malaria morbidity and mortality by reducing transmission. Parasitology Today 12: 236-240.

21. Grobusch MP, Egan A, Gosling R, Newman RD (2007) Intermittent preventive therapy for malaria: progress and future directions. Curr Opin Infect Dis 20: 613-620.
